# Advances in Detecting, Prognosticating, and Treating Hepatocellular Carcinoma: Advances and Outcomes

**DOI:** 10.3390/cancers16122165

**Published:** 2024-06-07

**Authors:** Ryan J. Kramer, Dimitrios Moris

**Affiliations:** 1 School of Medicine, Duke University, Durham, NC 27710, USA; rjk33@duke.edu; 2 Department of Surgery, Duke University Medical Center, Durham, NC 27710, USA

## 1. Introduction

Hepatocellular carcinoma (HCC) is the most common primary liver malignancy and the ninth leading cause of cancer-related death in the United States [1]. The most important risk factors for HCC include chronic viral infection, such as hepatitis B and C, and cirrhosis, related to alcohol ingestion, aflatoxin ingestion, and/or non-alcoholic fatty liver disease [1,2]. As these risk factors continue to increase in incidence and prevalence, the incidence of HCC has also increased [1], underscoring the need for improved detection and treatment.

The Barcelona Clinic Liver Cancer (BCLC) staging system has been established for prognosticating and directing treatment for HCC [3]. In early-stage disease (BCLC-0 or BCLC-A), local control therapies such as transplantation or hepatectomy are recommended when able and have a favorable five-year survival rate; however, this remains the case for the minority of patients [3]. Other therapies for intermediate or greater HCC include chemoembolization and systemic therapy, which are effective but nonetheless have limited survival [3].

This Editorial refers to the Special Issue “Advances in the Management of Hepatocellular Carcinoma”. The Special Issue highlights the advances in the management of HCC, focusing on various treatments.

Twenty-six manuscripts were submitted for consideration for the Special Issue, and all of them were subject to the rigorous *Cancers* review process. In total, twenty-three papers were finally accepted for publication and inclusion in this Special Issue (sixteen articles and seven reviews).

## 2. Overview of Articles Published in This Special Issue

### 2.1. Diagnosis, Risk-Prediction, and Pre-Therapy Prognostication

The cornerstone of all treatment is prompt diagnosis. This Special Issue presents several articles advancing the process of HCC diagnosis. **Contribution 1** leverages cell-free DNA as a mechanism of early diagnosis. The authors present a cohort of 27 patients with cirrhosis who had detectable cell-free DNA and MRI imaging available; they identified 6 patients who had precursor lesions and 2 patients who went on to develop lesions in follow-up. The cell-free DNA had 100% sensitivity, suggesting that cell-free DNA may be a useful adjunct to radiographic monitoring HCC development in liver cirrhosis.

Another subpopulation at risk of HCC that was studied in this Special Edition was those with chronic hepatitis B. **Contribution 2** presents a multicenter study validating the recent HCC prediction model FSAC in individuals with chronic hepatitis B starting antiviral therapy. Authors identify that, compared to other models, the integration of intra-hepatic fibrotic burden during antiviral therapy is essential and that FSAC outperforms other models in this subgroup. Another approach to patients with chronic hepatitis B infection receiving antiviral therapy was taken by **Contribution 3**, which presents a novel risk prediction algorithm specifically for this subpopulation. This manuscript generates three risk groups with statistically different HCC incidence, suggesting this may be a useful model for those with chronic hepatitis B on antiviral therapy.

Liver transplantation is one modality of surgical therapy in HCC that can establish local control. However, subsequent immunosuppression is necessary and can predispose to new primaries or HCC recurrence; thus, the post-transplant population presents a unique set of patients requiring surveillance and early diagnosis. **Contribution 4** analyzes germline variants in 334 patients with HCC considered for transplantation and 1,662 matched controls to identify putative genetic risk factors that might increase risk of malignancy following transplantation and immunosuppression. Interestingly, only 2.1% of those with HCC had identifiable pathogenic/likely pathogenic variants in the 226 gene panel, suggesting that the genetic contribution to HCC development is relatively minor but a select group of patients would benefit from this screening prior to transplantation. Specifically regarding HCC recurrence and mortality following liver transplantation, **Contribution 5** performs a meta-analysis and systematic review identifying 125 studies that demonstrated geographic variation in recurrence and survival. In all regions studied, however, the HCC recurrence incidence was at least 10% and with an overall 9% morality rate, suggesting that this problem remains significant.

The previous five articles all address problems of detection and diagnosis; however, the problem of prognostication is an important one both to patients and their care team. Microvascular invasion is a known risk factor in HCC recurrence following surgical therapy; however, it can only be confirmed post-operatively through pathology. **Contribution 6** uses a variety of preoperative metrics to identify predictors of microvascular invasion, finding that tumor volume and alpha-fetoprotein were independent risk factors. The authors develop a risk prediction model with moderate performance in prediction microvascular invasion that would be informative for treatment planning. **Contribution 7** leverages tumor burden score, a value that integrates tumor number and size, to stratify patients in the Scientific Registry of Transplant Recipients finding significant worse overall and recurrence-free survival among those with a higher tumor burden. Similarly, **Contribution 8** develops a prognostic model using tumor burden and albumin–bilirubin grade. The combination of these two factors provided successful predictive ability across HCC etiology, stage, and treatment strategy, suggesting that this user-friendly tool might be clinically applicable and useful.

### 2.2. Locoregional Therapy

There are multiple avenues for local control in HCC, ranging from surgical approaches like hepatectomy and transplantation to minimally invasive or non-invasive approaches like transarterial chemoembolization (TACE) or radiotherapy, or a combination of these approaches. Indeed, **Contribution 9** presents a systematic review and meta-analysis of randomized controlled trials studying hepatectomy alone versus hepatectomy with adjuvant TACE. Adjuvant TACE was associated with superior overall survival; however, these benefits have only been identified in Eastern Asian populations due to the absence of multinational trials. In further study of post-hepatectomy adjuvant therapy, **Contribution 10** compares TACE and intraoperative radiofrequency ablation. In this 617-person sample of individuals with intermediate-stage HCC and Child–Pugh class A liver function, those who received intraoperative radiofrequency ablation had significantly improved overall and progression-free survival versus those who received adjuvant TACE.

Both of the abovementioned studies compare adjuvant therapies following surgical resection; **Contribution 11** presents a comparison between surgical resection and TACE among elderly patients with BCLC-B stage HCC, finding that the population receiving surgery had a 97% three-year survival versus 32% in matched controls receiving TACE. The authors suggest that in carefully selected patients, surgery may be appropriate in those with BCLC stage B. Indeed, in the 242-patient Greek multicenter study, **Contribution 12** demonstrates that aggressive surgical management of HCC, including BCLC-B and BCLC-C HCC which comprised 29% of the cohort, produces offers impressive long-term survival with acceptably minimal short-term morbidity and mortality. Together, these four studies in this Special Issue suggest that there are many approaches to locoregional control; however, it appears that surgery is an important modality and there is substantial promise in adjuvant therapy, though more research is needed.

In addition to research comparing modalities, this Special Issue presents several articles that advance the methods of local control themselves. **Contribution 13** presents a pilot study leveraging a CT/MRI fusion imaging application in order to evaluate ablative margins in HCC radiofrequency ablation procedures. The authors find that the use of this application reclassified the initial imaging of seventeen individuals with local HCC progression despite initial categorization of complete response after radiofrequency ablation, suggesting this application is highly accurate and may be useful. Another avenue of research is in surgical approach to hepatectomy for HCC. **Contribution 14** presents a systematic review comparing robot-assisted hepatectomy to laparoscopic or open liver resection. The authors find that similar survival and comparable complication rate in all three approaches, suggesting equivalency. However, they underscore the need for non-industry driven randomized control trials to fully evaluate the role of robotic liver surgery, especially in patients with cirrhosis.

Because HCC is a relatively aggressive malignancy, substantial effort has been made in achieving locoregional control even in advanced disease. **Contribution 15** summarizes the treatment landscape for patients with portal vein tumor thrombus, which have advanced greatly in the past decade. The authors specifically highlight TACE and ablation as effective modalities, especially when combined with systemic therapies such as sorafenib. In patients with oligometastatic HCC, **Contribution 16** reports their 100-patient retrospective study KROG 20-04 which investigated the efficacy of stereotactic ablative radiotherapy. The overall survival rate was sixteen months and varied depending on performance status, Child–Pugh Classification, whether the primary tumor had been controlled, and the time interval of the metastases. Overall, these data support stereotactic ablative radiotherapy as a putative treatment in oligometastatic HCC.

### 2.3. Systemic Therapy

Systemic therapy is an essential component of therapy in advanced (BCLC-C) HCC. Sorafenib, a tyrosine kinase inhibitor, has been the cornerstone of systemic therapy; however, recent evidence has placed immune checkpoint inhibition as a first-line treatment. **Contribution 17** presents a systematic review and meta-analysis using real-world retrospective and prospective data including 38 studies. The authors find the best outcomes in combination therapy that includes immune checkpoint inhibition, tyrosine kinase inhibitors, and locoregional therapy. Interestingly, in a 127-patient retrospective cohort study presented in **Contribution 18**, authors find that traditional advanced HCC therapies (cisplatin-based chemotherapy, oral uracil–tegafur, surgery, radiation, and/or TACE) do not improve outcomes among those with advanced HCC who are also receiving tyrosine kinase inhibitors. These articles, heterogenous data, and diversity in the treatment landscape underscore the need for multicenter prospective trials studying combination therapies including multiple kinds of systemic and local therapies.

Even among those on the current first-line treatment modality for unresectable HCC, atezolizumab plus bevacizumab, there is substantial variation in response. To better prognosticate the response to therapy, **Contribution 19** studies neutrophil-to-lymphocyte and platelet-to-lymphocyte ratio among those on atezolizumab and bevacizumab. The authors found that these ratios were independent predictors of superior progression-free survival, suggesting these may be valuable prognostic methods and potentially relevant biologically. **Contribution 20** presents work optimizing the albumin–bilirubin grade for those with advanced (BCLC-D) HCC on a variety of anticancer therapies ranging from locoregional control (surgery, radiofrequency ablation, or TACE) to systemic therapy. The authors generate a modified albumin–bilirubin score that successfully predicts overall survival in HCC ranging from early to advanced disease.

### 2.4. New Frontiers in Hepatocellular Carcinoma Treatment

From the work presented in this Special Issue, it is clear that treatment modalities are continuing to evolve and progress in response to continued need. **Contribution 21** reviews the major developments and emerging therapies in HCC. Authors highlight the variety of locoregional and systemic therapeutic options currently present, and point to genetic and immunological therapies as safe and effective therapies for further exploration in HCC. Specifically, they discuss CAR-T cell therapy, immunotherapy, and gene therapy as prospective therapy in HCC. Similarly, **Contribution 22** discusses immunotherapy in HCC, diving into the translational research into the tumor microenvironment, the trials on mono- and combination immunotherapy in HCC, and future directions such as antibodies targeting TIM-3 and LAG3. Finally, **Contribution 23** presents important progress in translational HCC research, specifically the establishment of an immunocompetent rat model capable of studying both HCC primaries and metastases. This model demonstrates impressive promise in the study of HCC for biomarker derivation and therapeutic target testing in the future.

## 3. Conclusions

In conclusion, substantial progress has been made in the study of HCC. Research in improved detection and diagnosis, prognostication, local therapy, systemic therapy, and therapeutic target derivations provides a path forward in addressing this difficult malignancy. This impressive Special Issue highlights many of these advances made over the past few years alone, and provides a useful understanding of the current landscape of HCC.
